# Comparing effects of food mechanical properties on oral processing behaviors in two sympatric lemur species

**DOI:** 10.1002/ajpa.24809

**Published:** 2023-07-11

**Authors:** Nina Flowers, Mariana Dutra Fogaça, Haja Fabrice Razafindrabe Maminiaina, Jean Claude Razafimampiandra, Marlies Dolezal, Nayuta Yamashita

**Affiliations:** ^1^ Institute of Population Genetics University of Veterinary Medicine Vienna Austria; ^2^ Neotropical Primate Research Group – NeoPReGo São Paulo Brazil; ^3^ Department of Zoology and Animal Biodiversity University of Antananarivo Antananarivo Madagascar; ^4^ Platform for Bioinformatics and Biostatistics, Department of Biomedical Sciences University of Veterinary Medicine Vienna Austria; ^5^ Austrian Academy of Sciences Vienna Austria

**Keywords:** feeding behavior, functional morphology, primates, ring‐tailed lemurs, Verreaux's sifaka

## Abstract

**Objectives:**

The link between diet and the masticatory apparatus in primates is complex. We investigated how food mechanical properties (FMPs) and food geometry affect feeding behaviors and subsequent jaw loading. We compared oral processing in two sympatric lemur species with distinct diets and mandibular morphologies.

**Materials and Methods:**

All‐day focal follows of *Lemur catta* (*Lc*) and *Propithecus verreauxi* (*Pv*) were conducted in both the dry and wet seasons at Beza Mahafaly Special Reserve. We collected activity budget data, filmed feeding bouts, and collected food items to measure their mechanical properties with an FLS‐1 portable tester. Feeding videos for the top food items they spent the most time consuming were analyzed frame‐by‐frame to assess bite and chew numbers and rates.

**Results:**

*Lc* bite more and at a slower rate on tougher (maximum) foods, chew more for tougher (average) foods, and chew less for stiffer leaves. *Pv* initially increase chew number for tougher (average) foods, but their behavior is less affected as food toughness increases. *Pv* chew less and more slowly but spend more of the day feeding than *Lc*. Additionally, they have a tougher (maximum) diet than *Lc*.

**Discussion:**

*Lc* adjust their feeding behaviors depending on the FMPs of their top food items, while *Pv* feed more consistently. The more robust masticatory apparatus of *Pv* may not require them to adjust their feeding behaviors for more mechanically challenging foods. Furthermore, the two species show distinct differences in chewing. Exploring chewing on a daily scale could aid in understanding its impact on the loading of the masticatory apparatus.

## 1 INTRODUCTION

The relationship between primate diets and craniodental morphology has been studied for decades to understand primate evolution (Anapol & Lee, 1994; Bouvier, 1986a, 1986b; Daegling & McGraw, 2001; Hylander, 1979a; Laird et al., 2020; McGraw & Daegling, 2019; Ravosa, 1991; Taylor et al., 2008; Vinyard et al., 2007; Vogel et al., 2014; Wright et al., 2009; Yamashita, 2003). Clear correlations between dental morphology and diet have been found (Kay, 1975; Lucas, 2004; Lucas & Teaford, 1994; Strait, 1993a, 1993b; Taylor, 2002; Vogel et al., 2008; Yamashita, 1998, 2008a). Selection has shaped the masticatory apparatus to efficiently process specific diets (e.g., Bouvier, 1986a; Daegling, 1992; Daegling & Grine, 2006; Hylander, 1979a; McGraw & Daegling, 2019). During biting and chewing, stress and strain occur in the mandible as the muscles load the bone (e.g., Hylander, 1979b). Increasing bone mass or deepening the mandible have been suggested as adaptations to counter the bending loads occurring during chewing repetitions of challenging diets (Hylander, 1979a). However, direct relationships between diet and the masticatory apparatus have not been found consistently across primate species (e.g., Ross et al., 2012). As a result, the connection between diet and masticatory morphology continues to challenge researchers with its complexity.

Food items consumed by an animal are often described in qualitative categories, such as leaves, fruit, flowers, stalks, etc. While such categories are useful for describing geometry, they are not functional categories and assume homogeneity in both external and internal physical properties of food items (e.g., Coiner‐Collier et al., 2016; Taylor et al., 2008; Vogel et al., 2014). The physical properties can better be described both externally by their size and geometry and internally by their food mechanical properties (FMP), such as toughness and elasticity (Lucas et al., 1986; Talebi et al., 2016). Measuring diets by their FMPs instead of grouping them into broad qualitative categories (i.e., folivore, frugivore) was thought to be the missing link in the relationship between the masticatory apparatus and diets (e.g., Ross et al., 2012; Taylor et al., 2008; Vogel et al., 2014).

The amount and duration of the load that occurs during oral processing is determined in part by FMPs (e.g., Agrawal et al., 1998; Chew et al., 1988; Hylander, 1979a; Hylander & Johnson, 1994; Luschei & Goodwin, 1974; Oron & Crompton, 1985; Ross et al., 2007; Thexton et al., 1980; Vogel et al., 2014), and influences the stress and strain on the mandible and may result in adaptations of the mandibular morphology over time (e.g., Laird et al., 2020). Despite this link between oral processing and FMPs, clear relationships between FMPs and mandibular morphology have not been found, further calling into question the link between diets and masticatory apparatus (e.g., Norconk et al., 2009; Ross et al., 2012; Taylor et al., 2008; Vogel et al., 2014; Wright et al., 2009; Yamashita, 2002, 2003). Given the weak evidence for a unifying explanation, Ross et al. (2012) suggested that “mandibular morphology is only imprecisely related to diet” (p. 634). Further understanding the relationship between diets and the masticatory apparatus may therefore require a broader perspective. To address this, McGraw and Daegling (2019) asked not only to what degree do primate jaws reflect what is eaten but also how it is eaten.

Investigating how foods are orally processed may help further tease apart this complex relationship between the masticatory apparatus and diets (e.g., Kane et al., 2020; Laird et al., 2020; McGraw & Daegling, 2019; Ross et al., 2012). Oral processing begins with oral preparation of the food item, such as removing a fruit exocarp that is not consumed, followed by a bite‐off of the piece that will then be masticated and form the material into a swallowable bolus (Prinz & Lucas, 1995, 1997). The mandible carries loads throughout its length in all stages of feeding, so both biting and chewing need to be considered. Challenging diets may require greater bite forces or more repetitive mastication, producing stresses and strains that may be reflected in the masticatory apparatus (e.g., McGraw & Daegling, 2019; Ravosa, 1991; Ross et al., 2012). Moreover, both the frequency and duration of oral processing behaviors affect the loading of the masticatory apparatus (e.g., Laird et al., 2020; Ravosa et al., 2015). Greater data on food processing behaviors may, therefore, help further our understanding of the evolution of the masticatory apparatus.

In this study, we contrast feeding behaviors, both frequency and rate of biting and chewing, with respect to the mechanical properties and geometry of food items. We then used these more comprehensive descriptions of diet and their impact on behavior to compare two sympatric lemur species with similar body mass, distinct morphologies and dietary classifications (Schwartz et al., 1985; Tattersall & Schwartz, 1974). Verreaux's sifaka (*Propithecus verreauxi* (*Pv*); body mass ~2.8 kg) are folivores, with more than 50% of their diet consisting of leaf material (Richard et al., 2000; Yamashita, 2003, 2008b). Ring‐tailed lemurs (*Lemur catta* (*Lc*); body mass ~2.2 kg) are generalist herbivores that feed on fruits, flowers, and leaves (Sussman, 1991; Yamashita, 2003, 2008b). We explore their diets further by classifying them by their food geometry and material properties. The *Pv* dentition and gastrointestinal tract are consistent with that of a folivore; that is, developed crests and shallow basins on the molars and a spiral cecum (Campbell et al., 2000; Yamashita, 1998). *Lc*'s longer molar crests and elongated cecum are also more indicative of a folivorous diet, especially compared to other lemurids (Campbell et al., 2000; Yamashita, 1998). The mandibular morphologies of our study species are quite distinct, with *Pv* having a more robust mandible (e.g., a deeper mandibular corpus and ramus and a greater angle) associated with leaf‐eating (Hylander, 1979a; Ravosa, 1991; Schwartz et al., 1985; Selvey, 2018; Tattersall & Schwartz, 1974). The two species have very little overlap in their diets at both Beza Mafalay Special Reserve (BMSR) and Berenty Reserve (Simmen et al., 2003; Yamashita, 2002). Despite their different dietary classifications and distinct morphologies, previous research has not found a significant difference in the toughness of their diets (Yamashita, 2002, 2003). We explore this unexpected similarity in the toughness of their diets by classifying toughness on a finer scale using average toughness and maximum toughness. In addition, we consider the stiffness of the leaf materials consumed to investigate if this varies between the diets of the two lemur species.

We studied the impact of FMPs and food geometry on oral processing behaviors to better understand how the jaw is loaded in two sympatric lemur species with different diets and masticatory morphologies. Our analyses

1. looked at the impact of both the average and maximum toughness of all top food items and the membrane stiffness of leaf material on oral processing behaviors. We explore FMPs on a finer scale since previous research had not found the expected relationship between toughness and morphology (Coiner‐Collier et al., 2016; Taylor et al., 2008; Vogel et al., 2014; Yamashita, 2002, 2003). The link between FMPs and morphology may be an indirect relationship where FMPs impact oral processing behaviors which influence the stress and strain on the mandible. Variation in masticatory muscle activity during chewing sequences has been found to differ between primate species and to vary with FMPs (Vinyard et al., 2008). We hypothesized that tougher and stiffer food items would require more bites and chews to break down challenging plant materials (Ravosa et al., 2015; Yamashita, 2003). Biting and chewing tougher and stiffer plant materials were expected to take more time, leading to lower biting and chewing rates;
2. investigated the role that the size and geometry (hereafter referred to as “shape”) of food items had on oral processing. Since the geometry and size of food items can together restrict how foods can enter the oral cavity we created a shape variable to incorporate both characteristics into one (Table S1) (Laird et al., 2020; Yamashita, 2003). We expected larger food items to require more bites and chews and slower rates to break down as the size of an item can influence the load required to break it down (van Casteren et al., 2016).
3. compared the two lemur species' biting and chewing numbers and rates. Due to their distinct morphologies, we hypothesized that they would bite and chew food items differently due to differences in their masticatory apparatus. Previous behavioral observations noted that *Lc* appeared to feed faster than *Pv*, however, as this was the first attempt at quantifying their oral processing behaviors, we did not have clear expectations for how the two species would differ in biting and chewing a priori. Since we consider the rate of biting and chewing, we also looked at the time spent feeding for each species. In addition, because we looked at the impact of FMPs on oral processing, we compared FMPs (average toughness, maximum toughness, and membrane stiffness) between the two lemur species, both considering the food items each species spent the most time feeding on per season (top food items, Table S2) and their whole diets. We predicted that *Pv* would have a more mechanically challenging diet than *Lc* due to their more robust mandibular morphology. We hypothesized that this tougher diet would result in longer feeding times as suggested by Coiner‐Collier et al. (2016).

## 2 MATERIALS AND METHODS

### 2.1 Study site

This study occurred in Beza Mahafaly Special Reserve, a 4600‐hectare tropical dry forest in southwestern Madagascar (Ranaivonasy et al., 2016). Inside the reserve is an 80‐hectare semi‐fenced forest closed to human activity (Parcel 1) (Ranaivonasy et al., 2016). Parcel 1 transitions from a gallery forest in the east at the edge of the seasonal Sakamena River to a dry, deciduous forest to the west (Sussman & Ratsirarson, 2006).

### 2.2 Climate

Fieldwork was conducted in Parcel 1 during both the dry and wet seasons to sample the breadth of the annual diet. Dry season data collection occurred from May through July 2019 and wet season data from December 2019 through March 2020. More detailed climate information is in the supplementary materials S1. Total monthly rainfall data from the wet season fell well below Richard et al.'s (2000) mean monthly rainfall levels (December: −38.52%, January: −52.92%, and February: −25.15%) amassed from nearly 50 years of climate records (1945–1994) (Table S3). The % decrease in rainfall for three consecutive months compared to long‐term averages indicates meteorological drought conditions in our wet season sampling period (Ahmed & Razafison, 2021; Department of Water Resources, 2017; Global Drought Observatory, 2021a).

### 2.3 Data collection

We conducted 102 all‐day focal follows on habituated individuals of both lemur species to collect activity budget and diet data (Altmann, 1974). Seven lemur groups (4 *Pv* and 3 *Lc*) with overlapping territories throughout Parcel 1 were followed in each season. Within each social group, three to four distinguishable focal adult individuals were followed, selected based on the presence of a collar with a numbered pendant or unique, identifiable features. Both males (7 *Lc* and 9 *Pv*) and females (9 *Lc* and 6 *Pv*) were followed. Focal individuals were followed for 1 h at a time, rotating through all focals from the group throughout the day. Additional details on focal follow methods can be found in the supplementary materials S1.

During focal follows, data on time spent resting, moving, being social, and feeding were taken to create activity budgets for both lemur species. Feeding observations began when the food entered the oral cavity and ended when chewing finished. During feeding observations, data were taken on the plant species and part consumed. Feeding bouts were filmed ad libitum with a camcorder (Sony HDR‐PJ530, 9.2 megapixels or Panasonic HC‐V180, 10 megapixels) for more detailed post hoc data collection of biting and chewing that occurred too quickly in the field to quantify. Plants that were observed being fed on were flagged and parts from that plant or one nearby (matching size, location, and phenophase to those observed being eaten) were collected within 24 h of observation in sealed plastic bags with a small amount of water inside and kept out of the sun while they were transported back to the field laboratory. They were immediately photographed, measured, and tested. Taking into account both the geometry and size of each plant part, they were placed into four different shape categories to determine if plant shape influenced feeding behaviors. (Table S1).

Freshly collected plant parts were tested with a portable mechanical tester (FLS‐1) to measure toughness and membrane stiffness (Lucas et al., 2001, 2012). Tests were repeated on three different specimens of each plant part tested. Detailed methods for FMP testing are in the supplementary materials S1.

Two different toughness values were calculated per food item: the average and maximum toughness. Structural components (e.g., petioles and rachises) and outer coverings, such as fruit exocarp, comprised the maximum toughness. All tests done on these components for each given food item were averaged together to obtain its maximum toughness value. The remaining values, which included cuts through the lamina, midrib, and secondary veins for each given food item were averaged together to represent the average toughness.

Membrane stiffness was measured using a membrane test, which is a refinement of a blunt indentation test for the lamina of leaf material (Talebi et al., 2016; van Casteren et al., 2016). Instantaneous elastic modulus is a measure of the stiffness of the leaf lamina. Tests were repeated three times each per plant species and averaged together. Membrane stiffness was only measured on leaf material in the wet season.

Because bites and chews occurred too quickly to reliably count during focal observations, 315 feeding videos were analyzed to quantify and characterize bites and chews frame‐by‐frame (25 frames per second) in VideoLoupe (version 1.2.1; Corduroy Code Inc., 2018). Data were collected from the videos for each lemur species' top food items eaten in each season with the goal of watching 10 min of feeding sequence time for approximately 10 of the most frequently eaten foods (Table S2). Emphasis was put on total sequence time recorded instead of number of videos as their lengths varied. These foods were ranked according to the time spent feeding on them in focal follows. Detailed methods about which foods were analyzed are in the supplementary materials S1. Videos of 31 lemur individuals (10 *Lc* and 21 *Pv*) were selected based on quality but also to balance lemur sexes (Video numbers: *Lc*—81 F, 21 M; *Pv*—96 F, 115 M) focal groups, and months. Videos on males were limited for *Lc* due to fewer identifiable individuals being present.

A feeding sequence (=bout) consisted of any number of bites and subsequent chewing. The sequence began with biting, when the load was applied to the jaw as the lemur bit off a unit of food, included mastication, and ended when the load was released after the last chew. The unit was usually a single fruit or leaf. Time was recorded to the hundredth of a second. Data were taken per feeding sequence on the number and duration of bites and chews, total sequence time, and sequence time per unit of food consumed. Bites per second (bite rate) or chews per second (chew rate) were calculated by dividing the number of consecutive bites or chews by the amount of time in that period. All bite/chew variables were averaged for each video to control for different clip lengths.

### 2.4 Data analysis

Statistical analysis was done in R (version 4.1.1; R Core Team, 2021). We used a nominal significance cutoff of *p* < 0.05 after multiple testing correction; however, since we used a conservative approach, we report *p*‐values before and after adjustment. Plots were made using package ‘ggplot2’ (version 3.3.6; Wickham, 2016) using color package ‘Viridis’ (version 0.6.2; Garnier et al., 2021).

Linear mixed models were run using function lmer from package ‘lme4’ with option REML = F to request maximum likelihood estimation to test whether biting and chewing behaviors varied between lemur species (*Pv* and *Lc*), season (dry and wet), food shape, and FMPs for the top food items in each lemur species' diet (version 1.1.29; Bates et al., 2015). Sex and observer were included in the model as fixed effects to control for possible differences between males and females in biting and chewing behaviors and observer bias. In addition, to err on the side of caution, data from specific observers were excluded from certain models due to differences in data collection methods to prevent possibly biased and/ or confounded results. In total, 12 models were run with four different natural logarithm‐transformed response variables: average bite rate, average bite number, average chew rate, and average chew number, and three different Z‐transformed covariates: average toughness, maximum toughness, and membrane stiffness (Table S4). Season was included as a fixed effect for models with average toughness and maximum toughness; however, it was not included for models including membrane stiffness as a covariate because this was only collected in the wet season. In addition, tests of membrane stiffness were confined to leaf material so shape was limited to 2D1 and 2D2 for these models. Shape levels 3D1 and 3D2 were collapsed into one level, 3D, for models with average toughness as a covariate because there were not enough observations for these individual shape categories.

The interaction between FMPs and species was included in all models. In addition, due to differences in range for average and maximum toughness between the two lemur species, broken stick regressions were used to model the covariates in two parts, a lower range where values overlapped between the species and an upper range for *Pv* only. This was done to ensure that conclusions about the impact of average and maximum toughness on feeding behaviors were not drawn due to observations only from *Pv*. The cutoff between the overlapping and non‐overlapping sections of the species' ranges was made just beyond the last value for *Lc*.

In all models, lemur individual, and social group were included as random intercept effects to account for repeated observations. Finally, we implemented a maximal random slope structure to avoid over‐estimating fixed effects and to properly account for the complex co‐variance structure in our data (Barr et al., 2013; Osuna‐Mascaró et al., 2022; Schielzeth & Forstmeier, 2009). Details on the maximal random slope methodology are in the supplementary materials S1.

Residuals were checked for normality and homoscedasticity via quantile‐quantile plots and plots of residuals against fitted values to ensure that model assumptions were met. Random effects were checked for normality. Collinearity among fixed effects was checked on linear models with the same fixed effects as the corresponding linear mixed models used for hypothesis testing based on variance inflation factors (vif) calculated in the package ‘car’ (version 3.1.0; Fox & Weisberg, 2019; O'Hara et al., 2021). *p*‐Values for each fixed effect were obtained from the summary function in package ‘lmerTest’ (version 3.1.3; Kuznetsova et al., 2017). Multiple testing correction was applied across all four response variables that were tested with the same set of explanatory variables (see Table 1: models 1–4, models 5–8, models 9–12) using Bonferroni correction. Within the shape and observer variables for each model, all pairwise comparisons were calculated in the package ‘emmeans’ applying function ‘emmeans’ and ‘pairs’ (version 1.7.5; Lenth, 2022). These *p*‐values were corrected for multiple testing with Tukey's HSD post‐hoc test.

Kruskal–Wallis rank sum tests were used to determine if FMPs differed between the two lemur species' diets in both seasons (four level factor: *Lc—*Dry Season, *Lc*—Wet Season, *Pv—*Dry Season, *Pv*—Wet Season). Nonparametric tests were used for these comparisons because the data were not readily normalized. Membrane stiffness was only compared between species because data were only collected in the wet season. Pairwise comparisons were done with Wilcoxon rank sum tests, and the Bonferroni method was used to correct for multiple testing. In addition to comparing the FMPs of the top items in the two species' diets, we also compared the FMPs of all food items consumed during focal observations in both seasons. This whole diet comparison compared between the two lemur species the average toughness of 177 food items (70 *Lc*, 107 *Pv*), maximum toughness of 145 food items (63 *Lc*, 82 *Pv*), and the membrane stiffness of 57 food items (20 *Lc*, 37 *Pv*).

## 3 RESULTS

Our results for the different mixed models are presented in Table 1. We found no effect of sex or season in any of the models. An observer effect was found in some models (Table S6). This is discussed further in the supplementary materials S1.

### 3.1 Impact of FMPs on oral processing

#### 3.1.1 Average toughness

**FIGURE 1:**
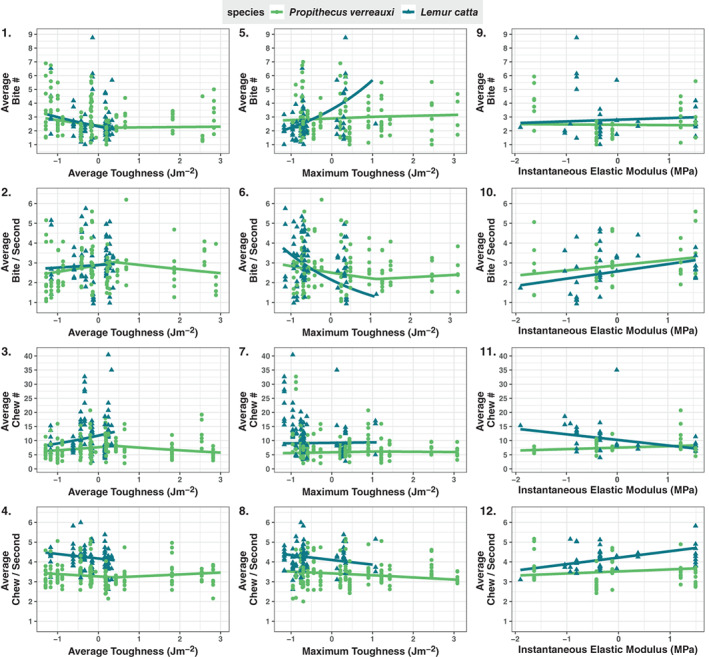
Relationship between food mechanical properties and oral processing. Lines represent mean model predictions. Points represent observational data scatter. Average toughness, maximum toughness, and instantaneous elastic modulus values are Z‐transformed. Response variables have been back‐transformed to reflect original values.

We found that average toughness (Table 1, Model 3: *R*_av_ < 0.4 adjusted *p*‐value = 0.079, *R*_av_ > 0.4 adjusted *p*‐value = 0.014) had an impact on chew number. Chew number increased as the average toughness increased (*R*_av_ < 0.4) indicating both lemur species chewed more for tougher (average) plant parts (Figure 1). *Pv* chew numbers declined as average toughness further increased (*R*_av_ > 0.04). This could suggest that *Pv* increase their chew number up until a certain toughness threshold and then begin to decrease chew number afterward (Figure 1).

**TABLE 1:** Model output of linear mixed models.

Model #	Response	Fixed effect	Estimate	SE	*p*‐Value	Bonferroni adjusted
1^a^	Bite number	*R*_av_ < 0.4	0.86	0.08	0.066	0.262
		*R*_av_ > 0.4	1.18	0.12	0.182	0.730
		Species	0.98	0.08	0.775	1.000
		Sex	1.05	0.06	0.442	1.000
		Season	0.92	0.12	0.493	1.000
		*R*_av_:Species	0.90	0.15	0.517	1.000
2^a^	Bite per second	*R*_av_ < 0.4	1.13	0.09	0.208	0.832
		*R*_av_ > 0.4	0.82	0.13	0.123	0.493
		Species	1.00	0.07	0.983	1.000
		Sex	0.93	0.05	0.153	0.611
		Season	1.09	0.08	0.327	1.000
		*R*_av_:Species	0.92	0.16	0.617	1.000
3^a^	Chew number	*R*_av_ < 0.4	1.17	0.07	0.020*	0.079
		*R*_av_ > 0.4	0.75	0.10	0.003**	0.014*
		Species	1.53	0.12	0.012*	0.048*
		Sex	1.05	0.05	0.344	1.000
		Season	1.33	0.14	0.114	0.454
		*R*_av_:Species	1.12	0.13	0.373	1.000
4^a^	Chew per second	*R*_av_ < 0.4	0.96	0.03	0.151	0.604
		*R*_av_ > 0.4	1.07	0.04	0.086	0.342
		Species	1.28	0.03	<2e−16***	8.00E−16***
		Sex	1.02	0.02	0.375	1.000
		Season	0.97	0.02	0.109	0.435
		*R*_av_:Species	0.98	0.05	0.712	1.000
5^b^	Bite number	*R*_max_ < 1.08	1.04	0.07	0.550	1.000
		*R*_max_ > 1.08	0.98	0.15	0.911	1.000
		Species	1.23	0.10	0.034*	0.134
		Sex	1.11	0.06	0.088	0.351
		Season	1.15	0.08	0.065	0.260
		*R*_max_:Species	1.53	0.12	0.001***	0.002**
6^b^	Bite per second	*R*_max_ < 1.15	0.88	0.06	0.030*	0.122
		*R*_max_ > 1.15	1.19	0.12	0.167	0.667
		Species	0.86	0.09	0.103	0.414
		Sex	0.94	0.05	0.281	1.000
		Season	0.90	0.07	0.126	0.504
		*R*_max_:Species	0.71	0.10	0.001**	0.005**
7^b^	Chew number	*R*_max_ < 1.1	1.04	0.08	0.656	1.000
		*R*_max_ > 1.1	0.94	0.14	0.718	1.000
		Species	1.57	0.11	0.002**	0.007**
		Sex	1.03	0.06	0.648	1.000
		Season	1.24	0.12	0.115	0.458
		*R*_max_:Species	0.98	0.14	0.871	1.000
8^b^	Chew per second	*R*_max_ < 1.08	0.97	0.02	0.208	0.832
		*R*_max_ > 1.08	0.99	0.04	0.877	1.000
		Species	1.20	0.03	1.69E−05***	6.76E−05***
		Sex	1.02	0.02	0.259	1.000
		Season	0.97	0.03	0.365	1.000
		*R*_max_:Species	0.97	0.04	0.389	1.000
9^c^	Bite number	*E*_inst_	0.99	0.08	0.894	1.000
		Species	1.15	0.12	0.245	0.980
		Sex	1.26	0.14	0.161	0.644
		*E*_inst_:Species	1.06	0.11	0.629	1.000
10^c^	Bite per second	*E*_inst_	1.10	0.06	0.143	0.572
		Species	0.88	0.11	0.232	0.928
		Sex	0.89	0.11	0.291	1.000
		*E*_inst_:Species	1.06	0.09	0.501	1.000
11^c^	Chew number	*E*_inst_	1.07	0.06	0.232	0.927
		Species	1.30	0.08	0.003**	0.011*
		Sex	1.04	0.09	0.651	1.000
		*E*_inst_:Species	0.77	0.08	0.001**	0.005**
12^c^	Chew per second	*E*_inst_	1.03	0.02	0.113	0.452
		Species	1.19	0.04	5.22E−04***	0.002**
		Sex	0.97	0.04	0.427	1.000
		*E*_inst_:Species	1.05	0.03	0.083	0.332

*Note*: *p*‐Values were generated with R package, lmerTest. Estimates are back‐ transformed to be ratios. The covariates (*R*_av_ and *R*_max_) are split based on the cutoff for the broken stick regressions (see text for further explanation). *R*_av_ = Z‐transformed average toughness; *R*_max_ = Z‐transformed maximum toughness; *E*_inst_ = Z‐transformed instantaneous elastic modulus. For pairwise results for shape see Table S5, and for observer see Table S6.

^a^ Models containing *R*_av_ as a fixed effect only have foods with an average toughness in the dataset. These models contain 266 observations of biting and chewing variables from 30 individuals in eight social groups.

^b^ Models containing *R*_max_ only have foods with a maximum toughness in the dataset. These models contain 266 observations of biting and chewing variables from 30 individuals in eight social groups.

^c^ Models containing *E*_inst_ only have plant material from the wet season in the dataset. These models contain 99 observations of biting and chewing variables from 23 individuals in seven social groups.

* *p* < 0.05 significance cutoff;

** *p* < 0.01;

*** *p* < 0.001.

#### 3.1.2 Maximum toughness

The interaction between maximum toughness and species was significant for bite number (Model 5: adjusted *p*‐value = 0.002) and bites per second (Model 6: adjusted *p*‐value = 0.005). *Lc* increased their number of bites (effect size = 0.468, 95% CI = [0.178, 0.758]) and decreased their bite rate (effect size = −0.461, 95% CI = [−0.710, −0.212]) as toughness (maximum) increased. *Pv* did not change their bite number (effect size = 0.040, 95% CI = [−0.162, 0.242]) or bite rate (effect size = −0.123, 95% CI = [−0.308, 0.063]) as foods got tougher (maximum).

#### 3.1.3 Membrane stiffness

The interaction between elastic modulus and species was significant for chew number (Model 11: adjusted *p*‐value = 0.005). *Lc* decreased their chew numbers on leaf material with a higher elastic modulus (effect size = −0.193, 95% CI = [−0.319, 0.005]), suggesting that they masticate less when breaking down stiffer leaf material (Figure 1). In contrast, *Pv*'s chew number was not influenced by elastic modulus (effect size = 0.0696, 95% CI = [−0.164, 0.304]).

### 3.2 Impact of shape on oral processing

The shape of the food item had an impact on the chew rate (Models 8 and 12). Pairwise comparisons of the estimated marginal means (emmeans) found significant differences between large leaves and small non‐planar foods (Table S5, Model 8: adjusted *p*‐value = 0.027). This indicates that large leaves are chewed more slowly than small fruits and flowers (Figure S1). Additionally, pairwise comparisons between small leaves and large leaves were significant (Table S5, Model 12: adjusted *p*‐value = 0.011), signifying that lemurs chew small leaves faster than large leaves (Figure S1).

### 3.3 Differences between lemur species

#### 3.3.1 Oral processing

The two lemur species significantly differed in their chew numbers (Model 3: adjusted *p*‐value = 0.048, Model 7: adjusted *p*‐value = 0.007, Model 11: adjusted *p*‐value = 0.011) and chew rates (Model 4: adjusted *p*‐value = 8.00 e^−16^, Model 8: adjusted *p*‐value = 6.76 e^−5^, Model 12: adjusted *p*‐value = 0.002). This indicates that in addition to chewing less, *Pv* also chews slower per feeding sequence than *Lc* (Figure 1). Additionally, as mentioned above, the two lemur species differed in their bite numbers with respect to the maximum toughness of the food item being orally processed (Model 5).

#### 3.3.2 FMPs

##### Average toughness

Average toughness of the top diet items of the two lemur species diets did not significantly differ (Table S7: adjusted *p*‐value = 1.00). When looking at the differences in average toughness of the whole diet, we found that the two lemur species' diets were significantly different in the wet season; *Pv* ate a tougher (average) diet than *Lc*, however, after multiple testing correction, the difference was no longer significant (Table S7: adjusted *p*‐value = 0.19; Figure S2).

##### Maximum toughness

The maximum toughness of the top diet items did not significantly differ between the two lemur species in either season (Table S7); however, both species' diets significantly differed between seasons with *Lc* and *Pv* eating tougher (maximum) top food items in the wet season (Table S7: *Lc* adjusted *p*‐value = 0.007, Table S7: *Pv* adjusted *p*‐value = 0.0117) than the dry season (Figure S2).

For the whole diets, we also found that *Lc* and *Pv* ate tougher maximum diets in the wet season (Table S7: *Lc* adjusted *p*‐value = 0.00159, Table S7: *Pv* adjusted *p*‐value = 0.00014) (Figure S2). The two lemur species did significantly differ in the maximum toughness of their whole diets in both seasons with *Pv* eating a tougher (maximum) diet than *Lc* (Table S7: wet season adjusted *p*‐value = 0.03682) (Figure S2). However, after multiple testing correction, the difference in dry season diets between the two lemur species was no longer significant (Table S7: adjusted *p*‐value = 0.166).

##### Membrane stiffness

The membrane stiffness of leaf material did not significantly differ in either the top food items (*χ* ^2^ = 0.43512, *df* = 1, *p*‐value = 0.5095) (Figure S2) or when comparing the whole diets in the wet season (*χ* ^2^ = 1.9502, *df* = 1, *p*‐value = 0.1626).

#### 3.3.3 Activity budget

**FIGURE 2:**
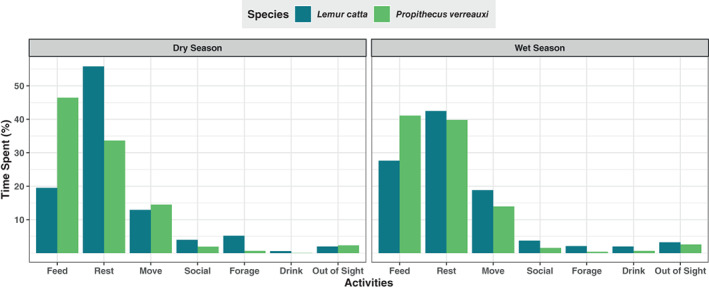
Activity budgets per season. The percentage of time spent is the amount of time spent in each activity divided by total follow time from sunrise to sunset.

*Pv* spent more time feeding than *Lc* in both the dry and wet seasons (Figure 2). *Lc* spent more time resting than *Pv* in both seasons. The difference between activity budgets between the two species was less pronounced in the wet season than in the dry season.

## 4 DISCUSSION

### 4.1 Impact of FMPs on oral processing

We expected both tougher and stiffer foods would require more bites and chews per bout and would take longer to orally process. Our results support this for some FMPs but not all. Average toughness and membrane stiffness did not significantly differ for bite number or rate (Table 1: Models 1, 2, 9, 10), and as with Ravosa et al. (2015), we found that none of the FMPs impacted chew rate (Table 1: Models 4, 8, and 12).

#### 4.1.1 Average toughness

We found that chew number increased with average toughness (Table 1: Model 3; Figure 1), indicating that lemurs may be compensating for tougher foods by increasing their number of chews per bout as expected. Interestingly, for *Pv*, the broken stick regression slope switched from positive to negative after reaching the toughness cutoff we designated based on the *Lc* average toughness range. This could suggest, for this lemur species, that up to a certain level of toughness (average), they increase their chew numbers, but once above a certain toughness (average) threshold, they slowly reduce their chew numbers. Since the amount of data after the cutoff for *Pv* is limited, trends for higher toughness (average) foods could be dependent on the cutoff value used. This should be considered when interpreting these results. However, for both species, we found that chewing (both number and rate) was not influenced by maximum toughness of plant parts. Taking the results for average and maximum toughness together, this suggests that the lemurs change their chewing behavior (chew number) initially as foods get tougher, but for sifakas this effect levels off as toughness continues to increase.

#### 4.1.2 Maximum toughness

We found that maximum toughness impacted both bite number and bite rate in *Lc*, but not in *Pv* (Table 1: Model 5 and 6; Figure 1). As foods get tougher (maximum), *Lc* bite more and at a slower rate, suggesting that they spent more time feeding on tougher (maximum) foods. We did not find the same trend in *Pv*. This could relate to differences in their masticatory morphology. With a more gracile morphology, *Lc* may need to adjust their feeding behaviors when consuming tougher (maximum) foods, whereas the greater robusticity of the *Pv* apparatus enables them to consume a wider range of tougher foods without altering their feeding behavior. One of the toughest (maximum) food items that *Lc* feed on is the tamarind fruit (though, interestingly, not the old fruit eaten in this study), a species whose origin has been questioned as it may have been introduced to Madagascar. If this is the case, it is possible that *Lc*'s masticatory apparatus has not evolved to deal with the mechanically challenging shell that encases the fruit, and *Lc* may adapt their biting behaviors to deal with this keystone resource in their diet. *Pv* may be increasing their bite force on tougher (maximum) foods instead of biting them more (Ravosa, 1991). These results are based on the top food items for each of the lemurs' diets in both the dry and wet seasons, which we found did not significantly differ in maximum toughness. With respect to their whole diets, *Pv* ate a significantly tougher (maximum) diet than *Lc*. While *Lc*'s top food items are comparable in toughness to *Pv*, the rest of their diet is not. Extending our investigation into daily biting and chewing frequencies instead of the per‐bout analysis only of top food items could clarify the differences in feeding behaviors between these two lemur species.

#### 4.1.3 Average and maximum toughness

Yamashita (2003) suggested that chewing, more than biting in lemurs, is impacted by the toughness of the food item, as tough foods need to be masticated to fragment them into fine particles and formed into a bolus to be swallowed. Yamashita (2003) describes average toughness as an “indicator of the loads that the masticatory apparatus bears during mastication” (p. 126) and maximum toughness as the “toughness of the foods where they are initially bitten off” (p. 126) when the plants are defending themselves against predators. In line with these ideas, we found that chew number was affected by the average toughness of food items and not their maximum toughness (Table 1: Model 3). Additionally, bite number and rate were impacted by the maximum toughness of food items but in *Lc* only.

#### 4.1.4 Membrane stiffness

Leaves with a low instantaneous elastic modulus (“membranes”) cannot retain their shape when a load is applied (Talebi et al., 2016). Alternatively, a higher instantaneous elastic modulus represents stiffer or more plate‐like leaves whose shape is held under a load (Talebi et al., 2016). We found that chew number was influenced by membrane stiffness for *Lc* only, which chewed less as leaf stiffness increased (Table 1: Model 12; Figure 1). One explanation for the negative relationship could be that, since stiffer leaves are more brittle, if enough force is applied, catastrophic failure breaks them into many smaller, more digestible pieces (Clissold, 2008; McGraw & Daegling, 2019). If this were the case, then both lemur species should have similar responses to membrane stiffness, yet *Pv* does not show much of a response. It could also be due to leaf size and the resulting volume of the mouthful that is then chewed; however incorporating intake data would be needed to investigate this further.

Leaves transition from being more membranous to stiffer as they develop (Talebi et al., 2016). Since less stiff leaves were chewed more by *Lc*, for this lemur species, younger leaves may actually require more chewing than mature leaves. When only considering the toughness of leaf materials, young leaves were believed to be chewed less since they are less tough than mature leaves (McGraw et al., 2015). However, McGraw et al. (2015) found that young leaves needed more chews than mature leaves in a leaf species consumed by red colobines. Here, we show that laminar stiffness also affects chew number in *Lc*, and that this may be tied to leaf maturity. Measuring membrane stiffness of thin flat materials provides new insights into feeding behavior for *Lc* that contrasts with assumptions about feeding behavior based on measuring toughness alone. This suggests that membrane stiffness could be an important element to further understand the link between diet, FMPs, oral processing, and the masticatory apparatus for species that rely on leaf material in their diets.

### 4.2 Impact of shape on oral processing

Coiner‐Collier et al. (2016) found that the major food categories (leaves, flowers, fruit, etc.) are internally inconsistent in their FMP values. Such food categories are a better representation of the food's geometry. Here we used food shape as a measure to incorporate both food geometry and size.

We found that differences in chew rate between small leaves and large leaves were only significant in models utilizing the reduced dataset with a limited number of shape observations (Model 12), indicating that this effect is likely related to a Type 1 statistical error (Figure S1). Additionally, since we collapsed our shape variable into more general categories for non‐planar shapes due to fewer shapes in the top food items, this may have precluded detection of any differences among non‐planar categories. Therefore, we can only confidently say that small leaves and large leaves did not affect biting and chewing (both number and rate). The lack of difference in biting between small and large leaves suggests that while a large leaf requires multiple bites per bout for the whole leaf to enter the mouth, lemurs may be biting multiple small leaves per bout before beginning to chew, suggesting that the volume of intake between small and large leaves is similar despite differences in leaf size. Future work will investigate intake volume and how it differs with food shapes and affects feeding behaviors between these lemur species (Hartstone‐Rose et al., 2015).

We still believe shape is an important variable to consider when investigating oral processing since it combines both the geometry and size of a food item. Food shape can influence placement of food items in different locations along the toothrow for ingestion (Yamashita et al., 2012). The location of where the load is applied can influence the stress and strain on the mandible, potentially influencing the morphology (Laird et al., 2020). We acknowledge the limitation of using only biting and chewing numbers and rate as proxies for loading instead of in vivo data on bone strain. However, our study complements experimental studies by examining how the animals deal with foods of different shapes/sizes and mechanical properties in the context of the full range of varied diets that these animals consume. Incorporating behavioral data collection on the location of initial bite‐off along the toothrow should therefore be an important consideration for studies on these lemur species.

### 4.3 Differences between lemur species

#### 4.3.1 Oral processing

We expected the two sympatric lemur species with distinct craniodental morphologies to process foods differently. We found the two lemur species differ in bite number and rate in relation to the maximum toughness of food items (Table 1: Model 5 and 6; Figure 1). In addition, the lemur species differ significantly in both chew number and rate in all our models (Table 1: Models 3, 4, 7, 8, 11, and 12; Figure 1). These differences in chew number and rate between the two species coincide with the known allometric effects that larger species chew less and more slowly (Druzinsky, 1993; Ravosa et al., 2010). *Pv* is slightly larger in body mass and has a more robust mandible (e.g., a deeper mandibular corpus and ramus and a greater angle) (Ravosa, 1991; Richard et al., 2000; Schwartz et al., 1985; Selvey, 2018; Sussman, 1991; Tattersall & Schwartz, 1974). Our findings emphasize that the differences in oral processing in the lemur species lie in chewing and their reactions to extreme food toughness (maximum) with *Lc* adjusting how they bite foods. Of relevance in this context are the greater robusticity of the mandible and associated structures (e.g., dental specializations, such as developed molar crests and shallow basins) that may have contributed to the relatively greater proficiency of *Pv* when eating certain foods (through *Lc* also possesses some traits related to folivory (e.g., long molar crests, elongated cecum) relative to other lemurids).

Wright et al. (2008) explored the chew rate between four folivorous colobines that consumed leaf material of similar toughness. The differences found in chew rate between species were attributed to differences in the masticatory efficiency of their post‐oral (digestive) processing. They suggested that chewing rate was related to how finely foods were masticated before swallowing, since the colobines that chewed more slowly and had larger particle sizes had an extra stomach chamber, the presaccus, to aid in further breaking down ingested food particles after swallowing. While the comparison is not exact, we also found that the lemur species with the lower chew rate (*Pv*) is a folivore with specialized gut morphology to help break down large quantities of leaf material that cannot readily form a bolus before swallowing (Lucas, 2004).

Since the two lemur species significantly differ in chew number and rate, we looked at the amount of time they spent feeding to understand their feeding strategies. We found that while *Pv* chewed less and more slowly per bout than *Lc*, they fed for a longer time seasonally (Figure 2). They may be compensating for chewing less, and thereby taking in less food per bout, by spending more time eating throughout the day to meet their daily dietary requirements. Comparing their daily bites and chews may help us to further understand differences in the feeding behaviors of the lemur species. Folivorous primates are expected to have more chewing bouts due to the quantity of leaf material in their diet, resulting in more repetitive stress during mastication (Ravosa, 1991). Given this, we would expect that *Pv* would chew more by the end of the day despite chewing less and more slowly per bout since they spend more time during the day feeding.

From an evolutionary perspective, Hylander (1979a) suggested that deeper mandibles are an adaptation to counter the loading caused by repetitive chewing of challenging diets. However, Ross et al. (2012) argued that chew number did not translate into fatigue failure since estimated strain magnitude (of human bone) would entail more chewing cycles than possible during the lifetime of the animal. While we did not measure or compare bone strain directly in our species, we note that we quantified increased stresses via foods of greater maximum toughness for *Pv* who also ate foods of a higher toughness than those eaten in common with *Lc*. The species with the more robust mandible chewed less per bout but spent more of the day feeding. We clearly need to look at daily chewing cycles to understand the impact of mastication on morphology. On balance from our findings here, repetition, in biting and chewing, likely played a role in shaping *Pv*'s morphology.

Another explanation for the difference in the masticatory apparatus between the two lemur species could be that *Pv*'s more robust mandible is driven by the resources that are most crucial in the diet (McGraw & Daegling, 2019). We investigated differences among FMPs between the two lemur species to explore this idea further.

#### 4.3.2 FMPs

We did not find differences in the FMPs of the top diet items between the lemur species (Table S7). Since this was a limited number of foods, we also compared the FMPs of their whole diets (Table S7). *Pv* had a tougher (maximum) diet than *Lc* throughout the year, although the dry season difference was no longer significant after multiple testing correction (Table S7, Figure S2). The species with a more robust mandible (*Pv*) has a tougher (maximum) diet, so the differences in the lemurs' masticatory apparatus could be related to the toughest (maximum) parts of their diets. Because maximum toughness did not significantly vary between lemur species for the top food items consumed but did when comparing the whole diet, the critical food items driving morphological differences may be eaten less frequently. *Pv*'s specialized morphology could have evolved as a result of feeding on foods that do not make up the bulk of the diet as suggested by Liem's paradox (Robinson & Wilson, 1998). Future research should look at feeding behaviors of less frequently eaten foods.

The two lemur species do not differ in the stiffness of the leaf material they consumed; however, the dataset for membrane stiffness was restricted to one season. Expanding the dataset to the dry season is needed to gain a more complete understanding of dietary membrane stiffness. Nevertheless, we found that membrane stiffness influenced chew number in *Lc* and not in *Pv*, suggesting that the two species may react differently to leaf materials and highlights the importance of collecting this type of data to differentiate species. Since our wet season FMP data were taken during a drought, additional data from a “normal” wet season would also allow us to see the impact the drought may have had on dietary FMPs.

### 4.4 Drought

The wet season data in our study were collected during drought conditions and the dry season data were also likely impacted as the drought spanned many years (Global Drought Observatory, 2021b). The lemurs' food choices in this study could have been affected by the effects of lack of rainfall on plant phenology and on the plant's mechanical properties. Previous research on the same population of ring‐tailed lemurs found leaf material made up 38% of their yearly diet (Yamashita, 2008b). The percentage of leaf material in the ring‐tailed lemurs' diets in this study was over 50% in both the dry and wet seasons, indicating a heavier reliance on leaf material that could be a result of the drought. Studying lemur diets during drought conditions may provide insight into future behavioral trends as climate change leads to more frequent droughts. Expanding on this study to incorporate wet season data from a non‐drought year would allow a comparison of normal season variation in feeding behaviors, FMPs, and time spent feeding in seasonal activity budgets. It would also allow a comparison of drought and non‐drought oral processing to further understand how climate change may impact the lemurs.

## 5 CONCLUSIONS

We found that *Lc* had a tendency to adjust their feeding behaviors for different FMPs, while *Pv*'s feeding behaviors remained largely unaffected. These differences in oral processing between the two species could be related to differences in their masticatory morphology. Additionally, *Pv* had a tougher maximum diet than *Lc*, which may be enabled by a more robust morphology that allows them to eat tougher foods without adjustment to their oral processing. We consistently found that *Pv* chewed less and more slowly than *Lc*, but spent more time feeding throughout the day. *Pv* fed more slowly but for longer than *Lc* who fed faster in less time. These key takeaways suggest that investigating daily biting and chewing may be important to understanding relationships among oral processing, FMPs, masticatory morphology, and diet.

## AUTHOR CONTRIBUTIONS

**Nina Flowers:** Conceptualization (equal); formal analysis (lead); investigation (equal); methodology (equal); visualization (lead); writing – original draft (lead). **Mariana Dutra Fogaça:** Conceptualization (equal); investigation (equal); methodology (equal); writing – review and editing (equal). **Haja Fabrice Razafindrabe Maminiaina:** Investigation (supporting); writing – review and editing (equal). **Jean Claude Razafimampiandra:** Investigation (supporting); writing – review and editing (equal). **Marlies Dolezal:** Formal analysis (supporting); methodology (supporting); validation (lead); writing – review and editing (equal). **Nayuta Yamashita:** Conceptualization (lead); data curation (lead); funding acquisition (lead); investigation (supporting); methodology (equal); project administration (lead); resources (lead); supervision (lead); validation (supporting); writing – review and editing (lead).

## CONFLICT OF INTEREST STATEMENT

The authors state that there is no conflict of interest.

## Supporting information


**Data S1.** Supporting information.

## Data Availability

The data that support the findings of this study are openly available in Zenodo at http://doi.org/10.5281/zenodo.7662076.
