# Supplementary material for: Comparing effects of food mechanical properties on oral processing behaviors in two sympatric lemur species
Source: Am J Biol Anthropol. 2023 Jul 11;182(1):45–58. doi: 10.1002/ajpa.24809 (PMC10952892; doi:10.1002/ajpa.24809)
Supplement: Supplementary file 1 — Data S1. Supporting information. [file AJPA-182-45-s001.docx]

**Supplementary Methods**

**Data Collection**

**Climate.** Southwestern Madagascar has a wet season from November to March and a long dry season from April to October (Sussman & Ratsirarson, 2006). During the dry season in this study, the maximum temperature was 36.0°C during the day and as low as 5.4°C at night. Total rainfall was 92 mm. During the wet season in this study, the maximum temperature was 49.2°C, and the minimum was 19.1°C with a total rainfall of 259.7 mm. The seasonal Sakamena River that borders the gallery forest on the eastern edge of Parcel 1 was not flowing for the majority of the 2019-2020 wet season

**Focal Follows.** Data were collected with continuous time focal animal sampling. Two teams of observers conducted focal follows six days a week, alternating days following *Lc* and *Pv*. Some focal individuals were only followed in one season, as they may have disappeared or switched groups. If this occurred a new individual of the same sex was followed. Focal groups were determined based on locations in Parcel 1 that maximized numbers of microhabitats. Each group was followed roughly once a week. The same four *Pv* groups were followed in the dry and the wet seasons. After our dry season data collection period, one of the *Lc* focal groups (Red group) disappeared. A new group was followed in the wet season (West group). Social groups ranged in total number of individuals both between groups and between seasons as males dispersed. Within this study, *Pv* group size ranged from three to seven individuals and *Lc* groups from four (Red group) to more than 20 individuals. Focal follows took place from the time the lemurs awoke in the morning to when they settled into their sleeping tree at night. Days were longer in the wet season due to the time of sunrise and sunset as well as the lemurs’ increased activity in the cooler morning and evening.

**FMP Testing.** The FLS-1 tester contains a load cell that measures the force applied to a sample and a linear variable displacement transducer (LVDT) that measures displacement. These signals are relayed to a laptop where software creates force-displacement graphs with the input of specimen dimensions to calculate mechanical values. Different tests are conducted depending on the shape of the specimen. All testing was conducted by NF and MDF.

Toughness was measured using either scissors or wedge tests. Both tests calculated toughness (R) as the work needed to fracture the item measured in J/m^2^. The test is done by applying a load to cut the item, propagating a crack. The friction of the cut is then subtracted from the test by running an empty pass. Scissors tests were used to cut thin food items such as leaves, stalks, fruit skins, and flowers. Leaf cuts were made at approximately 90 degrees to the midrib and included the lamina, secondary veins, and the midrib. Depending on the leaf size, cuts were made at the leaf’s base, middle, and tip. The wedge test was used for bulkier items such as fruits.

The membrane test measures the stiffness of flat items by applying a load for 10 seconds, holding it in place, and measuring the relaxation for 90 seconds. The load was applied with a probe to the lamina of a leaf. We use the instantaneous elastic modulus value rather than the infinite elastic modulus in our statistical models as it better represents the reaction of a plant part when a load is applied (van Casteren et al., 2016). Instantaneous elastic modulus is measured in MPa.

**Top Food Items.** The top foods eaten by the lemur species per season represented 71.2% (7 foods) and 72.8% (8 foods) of the total feeding time of the *Lc* diet and 60.5 + 4.1% (9 foods plus 2 foods eaten in common by the two species) and 48.6% (10 foods) of the *Pv* diet in the dry and wet seasons, respectively. In addition to the most frequently eaten foods, some foods that were commonly eaten by the two lemur species were also included. The majority of these were already included in the top foods eaten by both lemur species with the exceptions of “Mamyaho” leaves and old *Tamarindus indica* fruit that were frequently eaten by *Lc* but not *Pv*.

**Data Analysis**

**Maximal random slopes.** Random slopes were determined separately for each model. To be included as a random slope, a factor must have a minimum of two levels of random effect with two observations. A covariate needs to have, at a minimum, three unique values for each level per random effect. Random slopes for categorical effects were dummy coded and centered, and covariates Z-transformed, via a custom R script written by Roger Mundry (Schielzeth, 2010). Random intercepts and slopes were modeled as uncorrelated.

**Observer Bias**

Despite video observer training and comparisons, we still found an impact of observer for some models (Table S6). Some observer’s data were removed from the dataset when noticeable effects were found in the residuals. When collecting data to the hundredth of a second, small differences in data collecting can be compounded. Given this, it is important in such research to thoroughly check the diagnostic plots and properly account for observer bias in the statistical models, as done in this study.

|  |
| --- |

| **Table S1** | | | |
| --- | --- | --- | --- |
| *Plant shape codes for food items* | | | |
| Shape Code | Plant Type | Geometry | Size |
| 2D1 | Leaves | flat | Small |
| 2D2 | Leaves | flat | Large |
| 3D1 | Fruit / Flowers | non-planar | Small |
| 3D2 | Fruit / Stalks / Petioles | non-planar | Large |
| *Note*. Plant shape is a categorical variable that accounts for both the food item’s geometry and size. Small leaves measured less than 5cm in its longest dimension. Small fruits were less than 1.5 cm in diameter. | | | |

| **Table S2** | | | | | | | |
| --- | --- | --- | --- | --- | --- | --- | --- |
| *Top diet items for lemurs at Beza Mahafaly Special Reserve* | | | | | | | |
| Scientific Name (local name) | Plant Part Eaten | Shape | Total Sequence Time (mm:ss.ms) | Rav  (J/m^2^) | Rmax  (J/m^2^) | Einst  (MPa) | Time Sent Feeding (h:mm:ss) |
| *Lemur catta -* Dry Season | | | | | | | |
| (Mamyaho) | AL | 2D2 | 09:49.73 | 550.21 | 765.80 | NA | 6:06:32 |
| *Tamarindus indica* (Kily) | OldFr | 3D2 | 13:27.42 | NA | 853.89 | NA | 6:05:55 |
| *Metaporana parvifolia* (Kililo) | AL | 2D1 | 10:48.03 | 77.24 | 1033.02 | NA | 3:39:01 |
| *Tamarindus indica* (Kily) | YL | 2D1 | 34:05.17 | 407.34 | 877.78 | NA | 2:04:44 |
| *Talinella dauphinensis* (Dango) | AL | 2D1 | 11:15.11 | 324.04 | 991.86 | NA | 1:49:19 |
| *Ipomoea batatas* (Bageda) | AL | 2D2 | 11:04.07 | 356.18 | 528.20 | NA | 1:42:41 |
| *Tanulepis linearis* (Tamboro) | AL | 2D1 | 00:32.39 | NA | NA | NA | 1:22:14 |
| *Lemur catta -* Wet Season | | | | | | | |
| *Metaporana parvifolia* (Kililo) | AL | 2D1 | 10:10.54 | 417.54 | 2239.63 | 19.38 | 13:18:02 |
| *Talinella dauphinensis* (Dango) | RFr | 3D1 | 10:04.15 | 522.28 | 2186.45 | NA | 12:13:34 |
| *Tamarindus indica* (Kily) | YL | 2D1 | 09:45.43 | NA | 1071.48 | 21.29 | 4:34:20 |
| *Cedrelopsis grevei* (Katrafay) | YL | 2D2 | 10:27.95 | 261.69 | 1059.21 | 29.12 | 4:21:39 |
| *Pentopetio sp.* (Tsompia) | YL | 2D2 | 00:40.5 | 415.60 | 3079.22 | 18.86 | 2:28:22 |
| (Mamyaho) | AL | 2D2 | 07:08.33 | 522.65 | 1157.49 | 18.41 | 0:50:15 |
| *Propithecus verreauxi -* Dry Season | | | | | | | |
| *Acacia bellula* (Tratriotse) | YL | 2D1 | 12:24.64 | 35.40 | 2093.03 | NA | 10:46:38 |
| *Metaporana parvifolia* (Kililo) | AL | 2D1 | 17:07.61 | 77.24 | 1033.02 | NA | 9:59:12 |
| *Acacia bellula* (Tratriotse) | Fl | 3D1 | 05:17.97 | NA | NA | NA | 6:30:22 |
| *Talinella dauphinensis* (Dango) | AL | 2D1 | 13:36.34 | 324.04 | 991.86 | NA | 5:25:05 |
| *Grewia erythroxyloides* (Maintyfototse) | AL | 2D1 | 12:50.07 | 1409.01 | NA | NA | 5:23:34 |
| *Grewia grevei* (Kotipoke) | AL | 2D2 | 09:23.97 | 1071.73 | 1180.42 | NA | 4:51:59 |
| *Cedrelopsis grevei* (Katrafay) | AL | 2D2 | 10:30.91 | 683.77 | 1542.74 | NA | 3:33:14 |
| *Anacolosa pervilleana* (Tanjaka) | AL | 2D1 | 12:30.01 | 612.31 | NA | NA | 3:05:10 |
| *Euphorbia tirucalli* (Famata) | YSt | 3D2 | 10:01.09 | 526.92 | NA | NA | 3:02:52 |
| (Mamyaho) | AL | 2D2 | 02:50.90 | 550.21 | 765.80 | NA | 1:41:25 |
| *Tamarindus indica* (Kily) | OldFr | 3D2 | 18:26.60 | NA | 853.89 | NA | 1:39:36 |
| *Propithecus verreauxi -* Wet Season | | | | | | | |
| *Metaporana parvifolia* (Kililo) | AL | 2D1 | 13:43.15 | 408.50 | 2239.63 | 15.90 | 9:43:33 |
| *Dichrostachys humbertii* (Avoha) | YL | 2D1 | 10:07.48 | 412.98 | 5362.96 | NA | 9:06:52 |
| *Euphorbia tirucalli* (Famata) | YSt | 3D2 | 11:10.84 | 1315.98 | NA | NA | 6:18:32 |
| *Commiphora aprevalii* (Daro) | YL | 2D2 | 10:03.29 | 402.00 | 2878.23 | 27.89 | 4:56:01 |
| *Syregada chauvetiae* (Hazambalala) | UnFr | 3D1 | 07:10.67 | NA | 4639.56 | NA | 3:53:24 |
| *Terminalia mantaly* (Taly) | YL | 2D2 | 10:17.57 | 531.24 | 2352.68 | 21.00 | 3:34:18 |
| *Talinella dauphinensis* (Dango) | YL | 2D1 | 08:29.93 | 321.07 | 1350.44 | 22.29 | 3:28:34 |
| *Talinella dauphinensis* (Dango) | ML | 2D1 | 00:50.82 | 446.10 | 2287.97 | 22.29 | 0:37:11 |
| *Acacia rovumae* (Robonsty) | YL | 2D1 | 10:09.59 | 172.70 | 3476.43 | NA | 3:11:32 |
| *Acacia bellula* (Tratriotse) | YL | 2D1 | 09:55.91 | 133.88 | 3242.64 | NA | 2:32:09 |
| *Cedrelopsis grevei* (Katrafay) | YL P | 2D2 3D2 | 02:37.70 00:08.75 | 406.55 NA | 1059.21 | 29.12 NA | 2:11:32 |
| *Note.* Plant shape is a categorical variable that accounts for both the food items geometry and size together. R_av_, R_max_, and E_inst_ were tested separately for each lemur species but values were shared when applicable to fill in missing data per plant. AL = all leaves, YL = young leaves, ML = mature leaves, RFr= ripe fruit, UnFr = unripe fruit, OldFr = old fruit, Fl = flowers, YSt= young stalk, and P only = leaf petiole only. 2D1 = small flat, 2D2 = large flat, 3D1 = small non-planar, 3D2 = large non-planar. R_av_= average toughness, R_max_ = maximum toughness, and E_inst_ = instantaneous elastic modulus. | | | | | | | |

**Table S3**

| *Comparison of Monthly Precipitation with Long Term Averages* | | | |
| --- | --- | --- | --- |
| Month | 2019/2020 Monthly Rainfall (mm) ^†^ | Historical Average Monthly Rainfall (mm) ^‡^ | Decrease in Rainfall (%) |
| December | 87.00 | 141.51 | 38.52 |
| January | 82.10 | 174.38 | 52.92 |
| February | 84.58 | 113 | 25.15 |
| *Note.* The wet season at Beza Mahafaly Special Reserve is from November until March.  ^†^ Monthly rainfall data indicating drought conditions in the wet season 2019/2020. ^‡^ Compiled from 50 years of average monthly rainfall data from Richards et al. (2000). | | | |

| **Table S4** | | |
| --- | --- | --- |
| *Statistical Models for Analysis* | | |
| Model | Syntax |  |
| 1 | lmer(log(bite_number)~R_av*species+shape+sex+season+observer+  (1+R_av+sex.M+shape.2D2+shape.3D+season.dry\|\|group)+  (1+R_av+shape.2D2+shape.3D\|\|individual),data, REML=F) |  |
| 2 | lmer(log(bite_per_sec)~R_av*species+shape+sex+season+observer+  (1+ R_av+sex.M+shape.2D2+shape.3D+season.dry\|\|group)+  (1+ R_av+shape.2D2+shape.3D\|\|individual),data, REML=F) |  |
| 3 | lmer(log(chew_number)~R_av+species+shape+sex+season+observer+  (1+ R_av+sex.M+shape.2D2+shape.3D+season.dry\|\|group)+  (1+ R_av+shape.2D2+shape.3D+season.dry\|\|individual),data, REML=F) |  |
| 4 | lmer(log(chew_per_sec)~R_av *species+shape+sex+season+observer+  (1+ R_av+sex.M+shape.2D2+shape.3D+season.dry\|\|group)+  (1+ R_av+shape.2D2+shape.3D+season.dry\|\|individual),data, REML=F) |  |
| 5 | lmer(log(bite_number)~R_max*species+shape+sex+season+observer+  (1+R_max+sex.M+shape.2D2+shape.3D1+shape.3D2+season.dry\|\|group)+  (1+R_max+shape.2D2+shape.3D1+shape.3D2\|\|individual),data, REML=F) |  |
| 6 | lmer(log(bite_per_sec)~R_max*species+shape+sex+season+observer+  (1+R_max+sex.M+shape.2D2+shape.3D1+shape.3D2+season.dry\|\|group)+  (1+R_max+shape.2D2+shape.3D1+shape.3D2\|\|individual),data, REML=F) |  |
| 7 | lmer(log(chew_number)~R_max*species+shape+sex+season+observer+  (1+R_max+sex.M+shape.2D2+shape.3D1+shape.3D2+season.dry\|\|group)+  (1+R_max+shape.2D2+shape.3D1+shape.3D2+season.dry\|\|individual),data, REML=F) |  |
| 8 | lmer(log(chew_per_sec)~R_max*species+shape+sex+season+observer+  (1+R_max+sex.M+shape.2D2+shape.3D1+shape.3D2+season.dry\|\|group)+  (1+R_max+shape.2D2+shape.3D1+shape.3D2+season.dry\|\|individual),data, REML=F) |  |
| 9 | lmer(log(bite_number)~E_inst*species+shape+sex+observer+ (1+E_inst+sex.M+shape.2D2\|\|group)+(1\|individual), data, REML=F) |  |
| 10 | lmer(log(bite_per_sec)~E_inst*species+shape+sex+observer+ (1+E_inst+sex.M+shape.2D2\|\|group)+(1\|individual), data, REML=F) |  |
| 11 | lmer(log(chew_number)~E_inst*species+shape+sex+observer+ (1+E_inst+sex.M+shape.2D2\|\|group)+(1\|individual), data, REML=F) |  |
| 12 | lmer(log(chew_per_sec)~E_inst*species+shape+sex+observer+ (1+E_inst+sex.M+shape.2D2\|\|group)+(1\|individual), data, REML=F) |  |
| *Note*. This table displays the syntax for the mixed models in the R package, lme4. The response variables are natural logarithm-transformed and the covariates (R_av, R_max, E_inst) are z-transformed. The covariates (R_av and R_max) are split based on the cutoff for the broken stick regressions (see text for further explanation). Within the parentheses, random slopes are on the left side of the pipe and random intercepts on the right side. Uncorrelated random slopes and intercepts are represented by **\|\|**. | | |

| **Table S5** | | | | | | | | |
| --- | --- | --- | --- | --- | --- | --- | --- | --- |
| *Pairwise comparisons of the estimated marginal means (emmeans) for shape* | | | | | | | | |
| Model # | Response | Pairwise Comparison | Estimate | SE | DF | t.ratio | P-value | Tukey Adjusted |
| 1 | Bite Number | 2D1 / 2D2 | 1.10 | 0.16 | 11 | 0.63 | 0.540 | 0.806 |
|  |  | 2D1 / 3D | 1.20 | 0.17 | 8 | 1.27 | 0.240 | 0.448 |
|  |  | 2D2 / 3D | 1.09 | 0.19 | 13 | 0.51 | 0.617 | 0.866 |
| 2 | Bites Per Second | 2D1 / 2D2 | 1.06 | 0.17 | 12 | 0.34 | 0.738 | 0.938 |
|  |  | 2D1 / 3D | 0.86 | 0.16 | 8 | -0.86 | 0.415 | 0.679 |
|  |  | 2D2 / 3D | 0.81 | 0.18 | 16 | -0.96 | 0.354 | 0.615 |
| 3 | Chew Number | 2D1 / 2D2 | 0.91 | 0.09 | 25 | -0.89 | 0.381 | 0.650 |
|  |  | 2D1 / 3D | 0.85 | 0.25 | 8 | -0.57 | 0.582 | 0.838 |
|  |  | 2D2 / 3D | 0.93 | 0.28 | 9 | -0.25 | 0.807 | 0.966 |
| 4 | Chews Per Second | 2D1 / 2D2 | 1.03 | 0.03 | 9 | 0.90 | 0.390 | 0.652 |
|  |  | 2D1 / 3D | 1.00 | 0.05 | 11 | -0.09 | 0.930 | 0.996 |
|  |  | 2D2 / 3D | 0.97 | 0.05 | 9 | -0.64 | 0.538 | 0.802 |
| 5 | Bite Number | 2D1 / 2D2 | 1.06 | 0.12 | 10 | 0.47 | 0.650 | 0.964 |
|  |  | 2D1 / 3D1 | 1.23 | 0.25 | 5 | 1.02 | 0.360 | 0.750 |
|  |  | 2D1 / 3D2 | 0.64 | 0.24 | 4 | -1.20 | 0.291 | 0.655 |
|  |  | 2D2 / 3D1 | 1.16 | 0.27 | 9 | 0.65 | 0.534 | 0.914 |
|  |  | 2D2 / 3D2 | 0.60 | 0.23 | 5 | -1.32 | 0.243 | 0.589 |
|  |  | 3D1 / 3D2 | 0.52 | 0.22 | 7 | -1.55 | 0.163 | 0.459 |
| 6 | Bites Per Second | 2D1 / 2D2 | 1.14 | 0.19 | 11 | 0.79 | 0.448 | 0.858 |
|  |  | 2D1 / 3D1 | 0.78 | 0.18 | 7 | -1.08 | 0.319 | 0.713 |
|  |  | 2D1 / 3D2 | 1.57 | 0.25 | 4 | 2.77 | 0.054 | 0.162 |
|  |  | 2D2 / 3D1 | 0.69 | 0.19 | 13 | -1.34 | 0.202 | 0.554 |
|  |  | 2D2 / 3D2 | 1.38 | 0.29 | 11 | 1.53 | 0.154 | 0.452 |
|  |  | 3D1 / 3D2 | 2.01 | 0.56 | 10 | 2.50 | 0.031* | 0.118 |
| 7 | Chew Number | 2D1 / 2D2 | 0.83 | 0.07 | 9 | -2.32 | 0.047* | 0.167 |
|  |  | 2D1 / 3D1 | 1.29 | 0.31 | 8 | 1.06 | 0.320 | 0.722 |
|  |  | 2D1 / 3D2 | 1.07 | 0.49 | 7 | 0.15 | 0.885 | 0.999 |
|  |  | 2D2 / 3D1 | 1.54 | 0.38 | 10 | 1.76 | 0.111 | 0.349 |
|  |  | 2D2 / 3D2 | 1.29 | 0.59 | 7 | 0.55 | 0.602 | 0.945 |
|  |  | 3D1 / 3D2 | 0.83 | 0.43 | 10 | -0.35 | 0.731 | 0.984 |
| 8 | Chews Per Second | 2D1 / 2D2 | 1.07 | 0.03 | 14 | 2.34 | 0.035* | 0.138 |
|  |  | 2D1 / 3D1 | 0.88 | 0.04 | 5 | -2.81 | 0.041* | 0.133 |
|  |  | 2D1 / 3D2 | 1.17 | 0.14 | 4 | 1.25 | 0.280 | 0.635 |
|  |  | 2D2 / 3D1 | 0.82 | 0.04 | 7 | -3.73 | 0.007** | 0.027* |
|  |  | 2D2 / 3D2 | 1.09 | 0.14 | 4 | 0.71 | 0.516 | 0.889 |
|  |  | 3D1 / 3D2 | 1.33 | 0.18 | 6 | 2.18 | 0.070 | 0.227 |
| 9 | Bite Number | 2D1 / 2D2 | 1.12 | 0.20 | 15 | 0.66 | 0.521 | 0.521 |
| 10 | Bites Per Second | 2D1 / 2D2 | 1.03 | 0.13 | 9 | 0.27 | 0.793 | 0.793 |
| 11 | Chew Number | 2D1 / 2D2 | 0.91 | 0.11 | 10 | -0.82 | 0.430 | 0.430 |
| 12 | Chews Per Second | 2D1 / 2D2 | 1.16 | 0.06 | 14 | 2.94 | 0.011* | 0.011* |
| *Note.* Emmeans output results from pairwise comparisons within the fixed effect of shape. Plant shape is a categorical variable that accounts for both the food item’s geometry and size. Not all shapes were included in every model. 2D1 = small flat, 2D2 = large flat, 3D1 = small non-planar, 3D2 = large non-planar, 3D = non-planar.  * Indicates p< 0.05 significance cutoff. ** p< 0.01. ***p< 0.001. | | | | | | | | |

| **Table S6** | | | | | | | | |
| --- | --- | --- | --- | --- | --- | --- | --- | --- |
| *Pairwise comparisons for observer effect* | | | | | | | | |
| Model # | Response | Pairwise Comparison | Estimate | SE | DF | t.ratio | P-value | Tukey Adjusted |
| 1 | Bite Number | A / B | 0.98 | 0.07 | 168 | -0.29 | 0.772 | 0.772 |
| 2 | Bite Per Second | A / C | 0.86 | 0.05 | 176 | -2.55 | 0.012* | 0.012* |
| 3 | Chew Number | A / C | 1.17 | 0.07 | 232 | 2.53 | 0.012* | 0.033* |
|  |  | A / B | 1.37 | 0.09 | 225 | 4.92 | <.0001*** | <.0001*** |
|  |  | C / B | 1.17 | 0.08 | 222 | 2.23 | 0.027* | 0.068 |
| 4 | Chew Per Second | A / C | 0.98 | 0.02 | 262 | -0.82 | 0.415 | 0.693 |
|  |  | A / B | 0.95 | 0.02 | 245 | -2.04 | 0.043* | 0.106 |
|  |  | C / B | 0.97 | 0.03 | 245 | -1.15 | 0.251 | 0.485 |
| 5 | Bite Number | A / B | 0.97 | 0.07 | 160 | -0.45 | 0.655 | 0.655 |
| 6 | Bite Per Second | A / C | 0.84 | 0.05 | 192 | -2.95 | 0.004** | 0.004** |
| 7 | Chew Number | A / C | 1.21 | 0.08 | 237 | 2.92 | 0.004** | 0.011* |
|  |  | A / B | 1.50 | 0.10 | 236 | 5.95 | <.0001*** | <.0001*** |
|  |  | C / B | 1.25 | 0.09 | 223 | 2.97 | 0.003** | 0.009** |
| 8 | Chew Per Second | A / C | 0.95 | 0.02 | 251 | -2.19 | 0.030* | 0.076 |
|  |  | A / B | 0.93 | 0.02 | 243 | -3.01 | 0.003** | 0.008** |
|  |  | C / B | 0.98 | 0.03 | 238 | -0.87 | 0.386 | 0.660 |
| 9 | Bite Number | A / B | 1.01 | 0.13 | 61 | 0.04 | 0.966 | 0.966 |
| 10 | Bite Per Second | A / C | 0.94 | 0.10 | 72 | -0.62 | 0.535 | 0.535 |
| 11 | Chew Number | A / C | 1.28 | 0.13 | 67 | 2.51 | 0.015* | 0.015* |
| 12 | Chew Per Second | A / C | 0.86 | 0.03 | 91 | -4.61 | <.0001*** | <.0001*** |
|  |  | A / B | 0.90 | 0.03 | 91 | -3.11 | 0.003** | 0.007** |
|  |  | C / B | 1.05 | 0.04 | 92 | 1.30 | 0.196 | 0.397 |
| *Note.* Emmeans output results from pairwise comparisons within the observer fixed effect. All three observers were not included in every model.  * Indicates p< 0.05 significance cutoff. ** p< 0.01. ***p< 0.001 | | | | | | | | |

| **Table S7** | | | | | | | | |
| --- | --- | --- | --- | --- | --- | --- | --- | --- |
| *Wilcoxon Rank Sum Test Pairwise Comparisons of Food Mechanical Properties between Lemur Species* | | | | | | | | |
|  | Pairwise Comparison | Toughness (Average) | | Toughness (Maximum) | | | Instantaneous Elastic Modulus ^§^ | |
|  |  | P-value | Bonferroni Adjusted | | P-value | Bonferroni Adjusted | P-value | Bonferroni Adjusted |
| Top Food Items ^†^ | Lc: Dry - Wet | 0.25 | 1.00 | | 1.2 e^-3^** | 0.01* | - | - |
|  | Pv: Dry - Wet | 0.36 | 1.00 | | 2.0 e^-3^** | 0.01* | - | - |
|  | Dry: Lc - Pv | 0.32 | 1.00 | | 0.13 | 0.79 | - | - |
|  | Wet: Lc - Pv | 0.26 | 1.00 | | 0.06 | 0.34 | 0.56 | 0.56 |
| Whole Diet ^‡^ | Lc: Dry - Wet | 0.54 | 1.00 | | 2.7 e^-4^*** | 1.6 e^-3^** | - | - |
|  | Pv: Dry - Wet | 0.21 | 1.00 | | 2.4 e^-5^*** | 1.4 e^-4^*** | - | - |
|  | Dry: Lc - Pv | 0.20 | 1.00 | | 0.03* | 0.17 | - | - |
|  | Wet: Lc - Pv | 0.03* | 0.19 | | 0.01* | 0.04* | 0.17 | 0.17 |

^†^ Top Items refer to foods in each season on which the lemurs spent the most time feeding (see Table S2). ^‡^ The whole diet includes all food items (including top items) tested for mechanical properties in each season. Average toughness was calculated for 73 food items in the dry season and 104 items in the wet season. Maximum toughness was calculated for 64 food items in the dry season and 81 items in the wet season. ^§^ Instantaneous Elastic Modulus was only measured on leaf material consumed in the wet season. This included 57 food items.

* Indicates p< 0.05 significance cutoff. ** p< 0.01. ***p< 0.001

**Figure S1**

*Effect of Food Shape on Oral Processing*

**
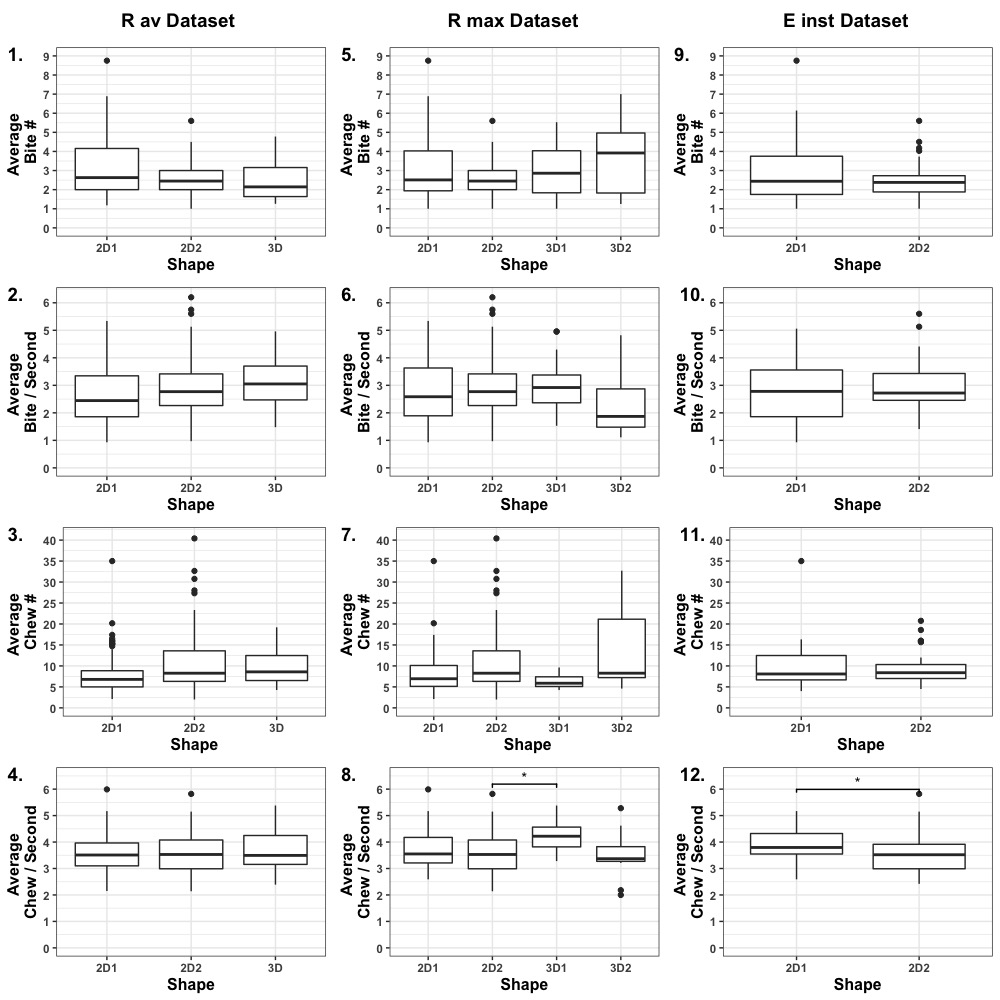
**

*Note.* Emmeans output results from pairwise comparisons within the fixed effect of shape. Plant shape is a categorical variable that accounts for both the food item’s geometry and size. Not all shapes were included in every model. Models containing Rav as a fixed effect only have foods with an average toughness in the dataset. Models containing Rmax only have foods with a maximum toughness in the dataset. Models containing Einst only have plant material from the wet season in the dataset. 2D1 = small flat, 2D2 = large flat, 3D1 = small non-planar, 3D2 = large non-planar, 3D = non-planar.

* Indicates p< 0.05 significance cutoff. ** p< 0.01. ***p< 0.001

**Figure S2**

*Comparisons of Food Mechanical Properties between Lemur Species*
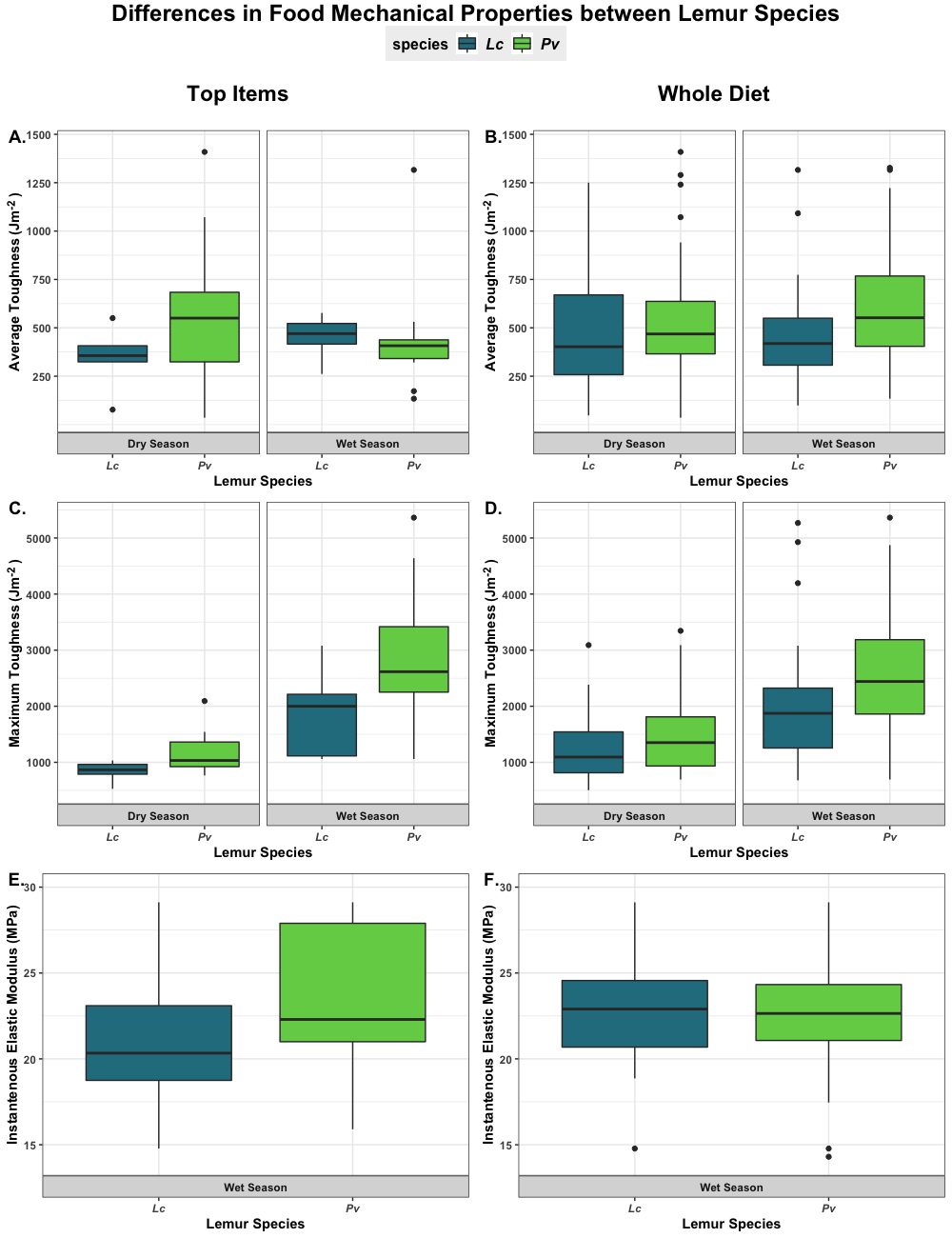


*Note*. Top Items (A, C, E.) refer to the mostly frequently eaten food items per season, see Table S2. The whole diet (B, D, F) refers to all food items (including top items) that were tested for mechanical properties in each season. Instantaneous Elastic Modulus was only measured on leaf material consumed in the wet season. Lc= *Lemur catta*, Pv= *Propithecus verreauxi*.

^†^ (A) n= 30, (B) n= 177, (C) n= 30, (D) n= 145, (E) n= 13, (F) n= 57.
